# Autoimmunomic Signatures of Aging and Age-Related Neurodegenerative Diseases Are Associated With Brain Function and Ribosomal Proteins

**DOI:** 10.3389/fnagi.2021.679688

**Published:** 2021-05-28

**Authors:** Junping Yin, Saleh Ibrahim, Frank Petersen, Xinhua Yu

**Affiliations:** ^1^Priority Area Asthma and Allergy, Research Center Borstel, Airway Research Center North (ARCN), Members of the German Center for Lung Research (DZL), Borstel, Germany; ^2^Institute of Experimental Dermatology, University of Lübeck, Lübeck, Germany; ^3^College of Medicine and Sharjah Institute for Medical Research, University of Sharjah, Sharjah, United Arab Emirates

**Keywords:** autoantibodies, autoimmunome, autoimmunomic signature, aging, age-related diseases, Parkinson's disease, Alzheimer disease, multiple sclerosis

## Abstract

Biological aging is a complex process featured by declined function of cells and tissues, including those of the immune system. As a consequence, aging affects the expression and development of autoantibodies and autoreactive T cells, which can be seen in their sum as the autoimmunome of an individual. In this study we analyzed whether sets of autoimmune features are associated with specific phenotypes which form autoimmunomic signatures related to age and neurodegenerative diseases. The autoantibody profile data of healthy subjects and patients from the GEO database was used to explore autoimmunomic signatures of aging and three neurodegenerative diseases including Parkinson's disease (PD), Alzheimer disease (AD) and Multiple Sclerosis (MS). Our results demonstrate that the autoimmunomic signature of aging is featured by an undulated increase of IgG autoantibodies associated with learning and behavior and a consistent increase of IgG autoantibodies related to ribosome and translation, and the autoimmunomic signature of aging are also associated with age-related neurodegenerative diseases. Intriguingly, Differential Expression-Sliding Window Analysis (DE-SWAN) identified three waves of changes of autoantibodies during aging at an age of 30, 50, and 62 years, respectively. Furthermore, IgG autoantibodies, in particular those against ribosomal proteins, could be used as prediction markers for aging and age-related neurodegenerative diseases. Therefore, this study for the first time uncovers comprehensive autoimmunomic signatures for aging and age-related neurodegenerative diseases.

## Introduction

As a complex biological process, aging develops with time on molecular, cellular, as well as on organ and organism's level. With age, the regenerative capacity of organs and tissues declines progressively and inevitably resulting in a step-wise loss of their physiological functions (Wyss-Coray, 2016). Beside physiological alterations, aging is also essentially associated with many pathological phenomena such as cancer and neurodegenerative diseases (Lopez-Otin et al., 2013). Intriguingly, the program of aging is partially encoded by circulating factors. This notion has been well-demonstrated by mouse heterochronic parabiosis models, where exposure to young blood leads to the rejuvenation of aging tissues such as brains, bone, heart, kidney, liver, pancreas and muscle (Conboy et al., 2005; Loffredo et al., 2013; Salpeter et al., 2013; Katsimpardi et al., 2014; Sinha et al., 2014; Baht et al., 2015). Furthermore, transfer of plasma proteins isolated from blood of young mice or human umbilical cord is able to revitalize brain functions in aged mice (Villeda et al., 2014; Castellano et al., 2017), suggesting a primary role of plasma proteins as the key circulating factor for the rejuvenation. In a recent study ~3,000 plasma proteins in more than four thousands healthy individuals were analyzed and a proteomic signature of aging was discovered (Lehallier et al., 2019). Interestingly, plasma proteins associated with age-related diseases are enriched in distinct waves of aging (Lehallier et al., 2019), suggesting a connection between aging and age-related disorders.

Natural autoantibodies are a specific group of plasma proteins which are present in all individuals (Coutinho et al., 1995). These autoantibodies are not pathogenic and participate in various physiological processes, such as immune regulation and tissue homeostasis (Lobo et al., 2010). Based on the enormous diversity of autoantibodies and autoreactive T cells and their individual expressions in response to genetic and environmental factors, their sum can be coined as “autoimmunome,” and an “autoimmunomic signature” represents a set of autoimmune features associated with a phenotype. In this study, we hypothesized that the autoimmunome is associated with physiological and pathological conditions. Among five main classes of immunoglobulins, IgG is the predominant class found in the circulation and it has the longest serum half-life (Schroeder and Cavacini, 2010). It has been demonstrated that natural autoantibodies of the IgG class are abundant and ubiquitous in human sera and their individual levels are affected by age, sex and disease (Nagele et al., 2013). Therefore, in order to explore and characterize the autoimmunomic signatures of aging and age-related diseases, we analyzed the profiles of IgG autoantibodies in healthy individuals as well as patients with early stage Parkinson's disease (ESPD), advanced stage Parkinson's disease (ASPD), Alzheimer disease (AD) and multiple sclerosis (MS).

## Methods

### Data Retrieval and Normalization

The dataset GSE62283 used in this study was retrieved from the NCBI GEO database. This dataset contains serum IgG autoantibody profiles of 156 healthy subjects, 103 patients with ESPD, 29 patients with ASPD, 54 AD and 30 patients with MS. In the current study, we excluded one healthy control subject who was 86 years since the big gap in age between this and the second oldest subject with 79 years disabled an analysis by DE-SWAN. Moreover, five samples from ASPD and eight samples from AD patients were excluded because of lacking information on person's age and/or sex. In total, the current study included serum IgG autoantibody profiles of 155 controls, 103 ESPD, 24 ASPD, 46 AD, and 30 MS samples. The demographic features of patients and healthy individuals are summarized in Supplementary Table 1. The autoantibody profile was determined as serum levels of IgG autoantibodies against 9,256 different full-length proteins.

The raw data of array of all subjects were downloaded and imported into R Studio (Version: 3.4.4) by using R based package limma. Raw files were first submitted to background correction using “backgroundCorrect (method=normexp),” and then “quantile” method was used to normalize the relative fluorescence unit among the arrays. After background correction and normalization, data of each array were log10 transformed and used for further analyses.

### Determination of Linear Changes in the Autoimmunome of Aging and Neurodegenerative Diseases

To determine autoimmunomic signatures in healthy subjects, we constructed following linear model with age (chronological age) and sex (male or female) as variants:

$$\begin{array}{l} levels\;of\;autoantibodies\;\sim \;\alpha +\beta 1age+\beta 2sex+\varepsilon \end{array}$$

To explore autoimmunomic signatures of neurodegenerative diseases, 103 ESPD patients, 24 ASPD patients, 46 AD patients, 30 MS patients and equal number of sex- and age-matched controls selected from the 155 healthy individuals were used for the analysis. To identify natural autoantibodies associated with each disease, we used the following linear model with diagnosis (patient or control), age (chronological age) and sex (male or female) as variants:

$$\begin{array}{l} levels\;of\;autoantibodies\;\sim \;\alpha +\beta 1diagnosis+\beta 2age+\beta 3sex+\varepsilon \; \end{array}$$

Coeffeciency and *p*-values (*F*-test) were calculated for each autoantibody. *P*-values were adjusted using method of Benjamini–Hochberg (BH). To determine the relative proportion of age and sex in explaining the change of natural autoantibodies, we output the type II sum of squares by using Anova function from package car in RStudio and calculated the partial Eta Squared (Eta2) using following formula:

$$\partial Eta2=\frac{\sum ofsquares_{effect}}{\sum ofsquares_{effect}+\sum ofsquares_{error}}$$

### Enrichment Analysis

To explore the enriched biological pathways and annotations, we utilized topGO package in RStudio (Version 3.4.4) and the DAVID online database (The Database for Annotation, Visualization and Integrated Discovery, version 6.8) which consists of a comprehensive collection of biological knowledgebase and functional analytic tools for understanding the biological meaning behind a list of genes or proteins. Significance of enrichment of GO (Gene Ontology), KEGG (Kyoto Encyclopedia of Genes and Genomes) pathways or UP-Tissue (Uniprot tissue) were determined using the 9,256 full-length human protein antigens on the protein array (www.invitrogen.com/protoarray, version 5.0), as the background list.

### Clustering of IgG Autoantibody Trajectories

To determine clusters of IgG autoantibodies, log10 transformed levels of each autoantibody were z scaled and were fitted using locally weighted scatterplot smoothing (LOESS) regression, which is a regression technique utilized to characterize the relationships between paired features by fitting a smooth curve to the scatter plot of data points. To classify each autoantibody into similar group, the distance between each pair of autoantibodies was calculated by the Euclidian method and the complete method was used for hierarchical clustering. To further understand the biological pathways and functions of each cluster, we performed enrichment analysis as described above.

### Differential Expression–Sliding Window ANalysis (DE-SWAN)

To explore changes of natural IgG autoantibodies during aging, we utilized the DE-SWAN analysis tool which was recently established by Lehallier et al. for identification of linear and non-linear changes during aging (Lehallier et al., 2019). This tool analyzes levels of autoantibodies within a window of 20 years and compares two groups in parcels of 10 years (e.g., 30–40y compared with 40–50y), while sliding the window in increments of 1 year from young to old (Lehallier et al., 2019). By virtue of DE-SWAN, we identified significant changed natural autoantibodies at each year with a threshold of an adjusted *p*-value (method = BH) lower than 0.05. Age was binarized according to the parcels and the formula of linear model that we used in the DE-SWAN is as follows:

$$\begin{array}{l} levels\;of\;autoantibodies\;\sim \;\alpha +{\beta 1age}_{\frac{low}{high}}+\beta 2sex+\varepsilon \end{array}$$

### Prediction of Aging and Aging-Related Diseases by Using IgG Autoantibodies

To estimate whether natural autoantibodies could be used for prediction of aging and aging-related diseases, we utilized the elastic net algorithm which is a popular regularized regression algorithm and commonly used in exploration of predictive signatures (Lehallier et al., 2016). For classification of aging, all 155 healthy subjects were divided into two groups, one including subjects whose age were below 65 years and a second group including subjects whose age were equal to or higher than 65 years. For classification of aging-related diseases, 103 ESPD patients, 24 ASPD patients, 46 AD patients, 30 MS patients and equal numbers of sex- and age-matched healthy controls were included in the analysis. For each classification trial, the dataset was randomly divided into two parts: a training set which contains 2/3 of the data and a testing set which contains the residual 1/3 of data. An elastic net (alpha = 0.8, 100 lambda tested, “lambda.min” estimated after 10-fold cross validation was used as the shrinkage variable) was built using the training data set. In order to minimize the error of autoantibodies estimated due to random sampling, we performed 500 times iterative resampling of training and testing set. For each of permutation, average of accuracy, sensitivity and specificity were calculated. Standard deviation of accuracy across 500 permutations was used to estimate the stability of model. A lower standard deviation of accuracy indicates higher stability of the model. Besides, to evaluate the importance of autoantibodies in the prediction and selected top autoantibodies, we ranked the autoantibodies according to the number of appearances in elastic net model across 500 permutations and termed importance index.

To further reduce the autoantibody used in the predictive model to reach optimal accuracy, sensitivity and specificity, we ranked the autoantibodies according to the importance index and select top 2, 5, 10, 15, and 20 important autoantibodies for classification of aging and aging-related diseases. A ridge regression model (alpha = 0, 100 lambda tested, “lambda.min” estimated after 10-fold cross validation was used as the shrinkage variable) was built using top 2, 5, 10, 15, and 20 autoantibodies. Again, 500 times iterative resampling of training and testing set was performed. Averages of sensitivity and specificity, average and standard deviation of accuracy across 500 permutations were estimated.

## Results

### Linear Modeling Reveals an Autoimmunomic Signature of Aging

**Figure 1:**
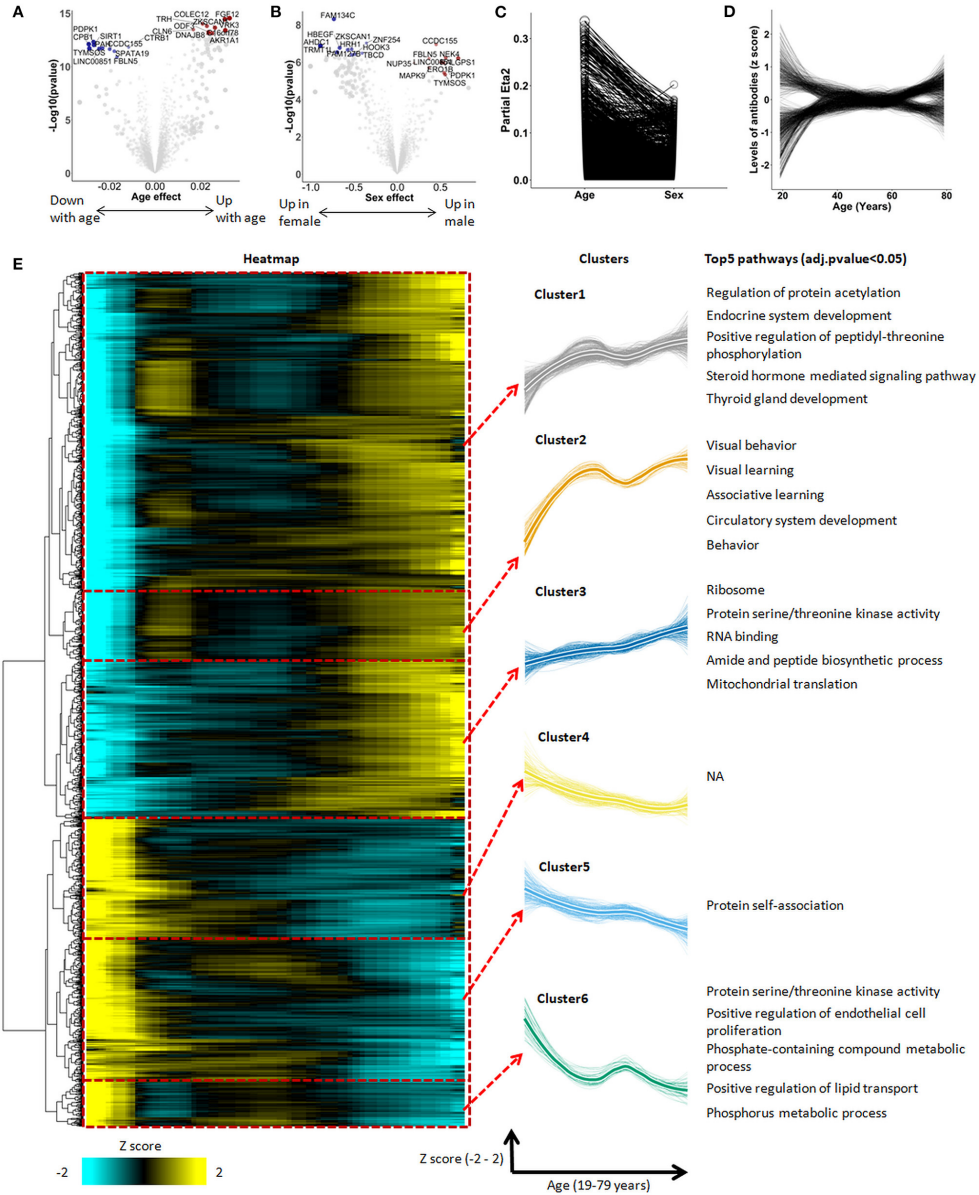
The autoimmunomic signature of aging identified by linear modeling. In total, serum levels of IgG autoantibodies against 9,256 human antigens were analyzed using linear models. Volcano plots representing changes of the autoimmunome with sex **(A)** and age **(B)**. Dot sizes are proportional to the product of –log10(*p*-value) and sex or age effect (beta of the linear model). **(C)** Relative percentage of variance explained by age and sex, where partial Eta2 is calculated for age and sex. Values for each autoantibodies are connected by edges. **(D)** Autoantibody trajectories during aging. Levels of 1,276 aging-associated IgG autoantibodies were z-scored and trajectories of the autoantibodies were estimated by LOESS. **(E)** Heatmap and unsupervised hierarchical clustering of trajectories of the 1,276 aging-associated autoantibodies. The heatmap was used to group autoantibodies with similar trajectories. The right panel shows the 6 clusters, with thicker lines representing the average trajectory for each cluster. The top 5 significantly enriched pathways are represented for each cluster. Pathway enrichment was tested using GO and KEGG databases.

To determine a potential relationship between aging and the autoimmunome, we retrieved dataset GSE62283 from GEO database and analyzed serum IgG antibodies against 9,256 different autoantigens in 155 healthy subjects ranging in age between 19 and 79 years (DeMarshall et al., 2015). Using linear modeling, we first accessed autoimmunomes of aging and sex. Within the IgG autoantibodies analyzed, 1,276 of them showed significant (*q* < 0.05, Figure 1A) alterations depending on age, with a strongest effect seen on autoantibodies against vaccinia related kinase 3 (VRK3), fibroblast growth factor 12 (FGF12), thyrotropin releasing hormone (TRH), collectin subfamily member 12 (COLEC12), and zinc finger with KRAB and SCAN domains 1(ZKSCAN1). Regarding sex, levels of 238 IgG autoantibodies were found to be significantly different between women and men (*q* < 0.05, Figure 1B). Interestingly, the majority of autoantibodies associated with the sex (194 of 238) also display significant changes during aging (Supplementary Table 2). However, effects of age on autoantibody expression exceed those of sex not only with regard to the number of different autoantibody affected but also to their individual differences in the expression level (Figure 1C).

When visualized as z-scored changes, the 1,276 IgG autoantibodies significantly associated with aging could be categorized by two groups with a first group containing 816 autoantibodies increasing during aging and a second group of 460 autoantibodies which levels decreased over age (Figure 1D, Supplementary Table 3). All significant aging-related IgG autoantibodies were further grouped into six clusters by unsupervised hierarchical clustering (Figure 1E, Supplementary Table 4). Beside four clusters showing an undulating pattern (clusters 1, 2, 5, and 6), clusters 3 and 4 changed linearly over age.

To explore the biological relevance of these changes, we performed enrichment analysis for antigens targeted by aging-associated IgG autoantibodies using Database for Annotation, Visualization and Integrated Discovery (DAVID) which provides a set of data-mining tools that promote the discovery of gene ontology (GO) functional categories. Based on this analysis, 5 out of the 6 clusters were significantly enriched for specific biological terms (*P*_adjusted_ < 0.05, Figure 1E, Supplementary Table 5). Interestingly, IgG autoantibodies against antigens enriched in ribosome, translation, mitochondrial translation and mitochondrial ribosome were found to consistently increase during aging (cluster 3). Moreover, autoantibodies directed against antigens enriched in behavior, learning, fatty and lipid oxidation increased with age in an undulating manner (clusters 1 and 2), while those directed against antigens enriched in endothelial cell proliferation and protein phosphorylation decreased during aging (cluster 6).

### Waves of Changes of IgG Autoantibodies During Aging Revealed by Quantifying Autoimmunomic Changes

**Figure 2:**
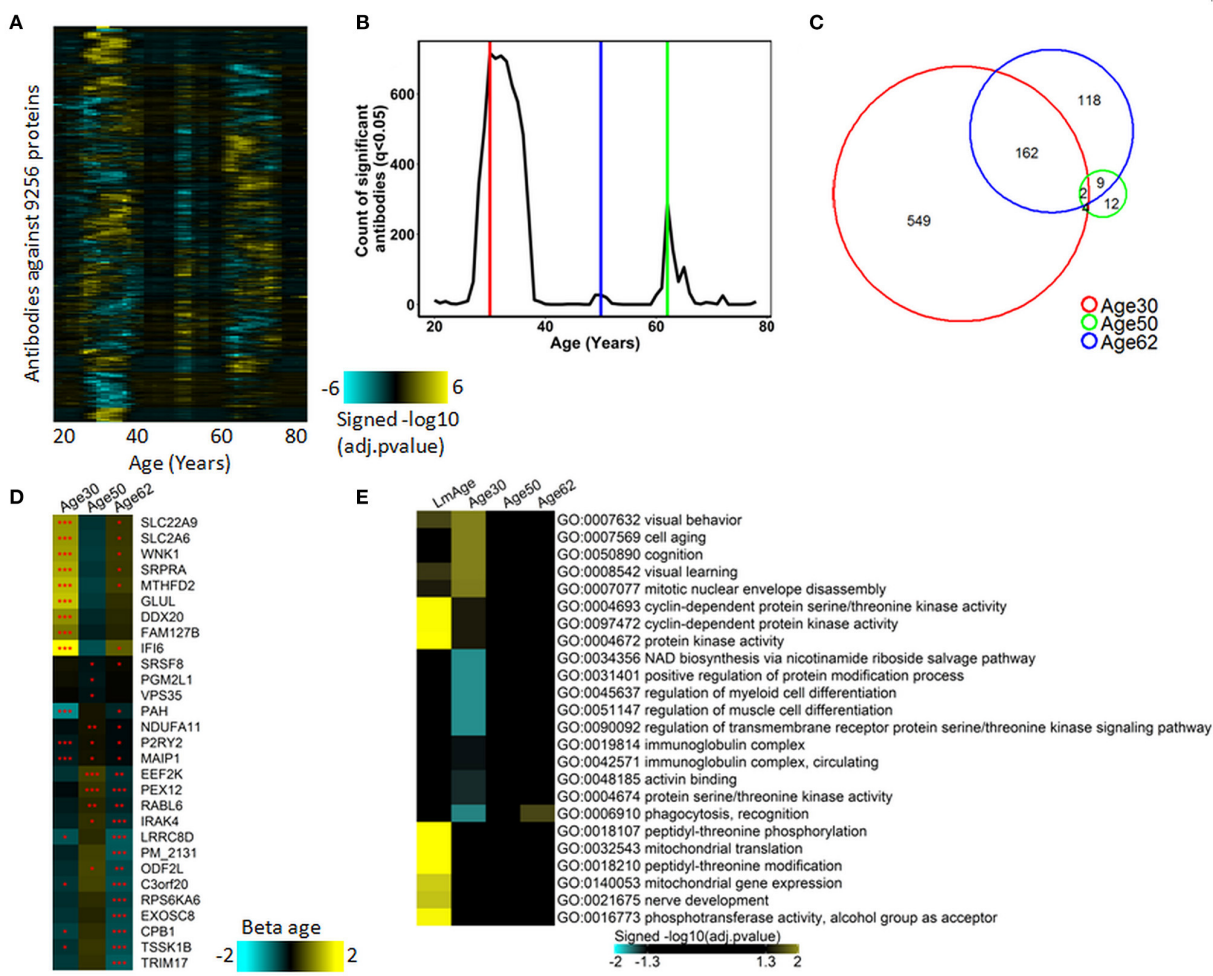
Waves of autoimmunomic changes with age. **(A)** Changes in autoantibody expression during aging in 155 healthy subjects. Within each window, *p*-values plotted in a log10 scale were estimated by DE-SWAN with age and sex as covariates and then adjusted using the Benjamini–Hochberg method. **(B)** Number of autoantibodies differentially expressed (*P*_adjusted_ < 0.05) during aging in ESPD patients identified by DE-SWAN. Three waves peaking at the age of 30 (red line), 50 (blue line), and 62 (green line) years, respectively, were identified by using DE-SWAN. **(C)** Overlaps among aging-associated autoantibodies identified by DE-SWAN at the age of 30, 50, and 62 years (*P*_adjusted_ < 0.05). **(D)** Top 10 significanlty changed autoantibodies identified by DE-SWAN at the age of 30, 50, and 62 years, respectively. Blue and yellow colors represent local increase and decrease, respectively (*P_adjusted_ < 0.05, **P_adjusted_ < 0.01, and ***P_adjusted_ < 0.001). **(E)** Visualization of pathways significantly enriched for aging-associated autoantibodies identified by linear modeling (LmAge) and DE-SWAN at the age of 30, 50, and 62 years. *p*-values were calculated by DAVID server using Fisher's test and adjusted using the Benjamini–Hochberg method. Autoantibodies upregulated and downregulated were analyzed separately. Gene ontology (GO) terms and KEGG pathways are shown, and the top 10 significant pathways per condition are represented.

Previously, Lehallier et al. developed a software tool termed differential expression-sliding the window analysis (DE-SWAN) and utilized it for the quantification of the proteomic changes across the lifespan (Lehallier et al., 2019). To better characterize the autoimmunomic signature of aging, we applied the DE-SWAN to quantify the changes of IgG autoantibodies. As shown in Figures 2A,B, the determination of differentially expressed (*P*_adjust_ < 0.05) autoantibodies uncovers three waves of changes of autoantibodies at an age of 30, 50, and 62, years, which contains 717, 27, 291 IgG autoantibodies, respectively (Supplementary Table 6). Noticeably, there were considerable overlaps in the composition of the three aging-related waves (Figure 2C). For example, 7 out of top 10 differentially expressed autoantibodies in the wave at age 30 were present also in the wave at age 62, and 8 out of top 10 differentially expressed autoantibodies in the wave at age 50 also showed up in the wave at age 62 (Figure 2D). Two autoantibodies, anti-purinoceptor 2 (P2RY2) and anti-matrix AAA peptidase interacting protein 1 (MAIP1) were consistently present in all three waves.

To further characterize autoantibodies significantly changes in the three waves, we performed enrichment analysis using DAVID. The result demonstrated that IgG autoantibodies upregulated in the peak of 30 years were enriched in GO terms of visual behavior, cell aging, cognition and learning, while those downregulated at the peak of 30 were enriched in GO terms of regulation of cell differentiation, cell migration, cell motility, localization of cell and protein activation cascade. With autoantibodies changes in the waves of 50 and 62 years, the only significantly enriched GO term was phagocytosis, recognition for upregulated autoantibodies in wave 62 year (Figure 2E, Supplementary Table 7).

Previously, Lehallier et al. identified three aging-related waves of plasma proteins (Lehallier et al., 2019). To explore the relationship between autoimmunome and plasma proteome, we compared the three aging-related waves of autoantibodies identified here and aging-related waves of plasma proteins identified by Lehallier et al. Interestingly, only very limited number of autoantigens were observed in both groups (Supplementary Figure 1A), suggesting that the production of IgG autoantibodies is unlikely associated with that of plasma proteins. Based on this finding, we hypothesized that the expression of serum IgG autoantibodies could be associated with the presence of autoantigens in tissues and organs. To validate this notion, we performed enrichment analysis for aging-related waves of autoantibodies using the DAVID tool to discover their relevant UP-TISSUE categories. Intriguingly, proteins targeted by autoantibodies in the wave at 62 years were enriched in thyroid, thalamus, uterine endothelium and several types of tumors such as dermoid, rectum and esophagus tumor, while those targeted by autoantibodies in the wave at 30 years were enriched in hippocampus, placenta and muscle (Supplementary Figure 1B).

To investigate whether the aging-related waves of autoantibodies also exist under pathological conditions, we utilized the DE-SWAN tool to analyze the autoimmunome of 103 patients with ESPD in an age range between 37 and 79 years. In contrast to healthy subjects, no aging-related wave of autoantibodies was observed in patients with ESPD (Supplementary Figures 2A,B), suggesting that the disease might have a strong impact on the autoimmunome which masks the effect of aging. This notion was supported by the finding from linear modeling analysis with ESPD patients and healthy controls, where the disease showed a significantly stronger effect on autoantibodies as compared to both, age and sex (Supplementary Figure 2C).

### Autoimmunomic Signatures of Aging-Related Neurodegenerative Diseases

**Figure 3:**
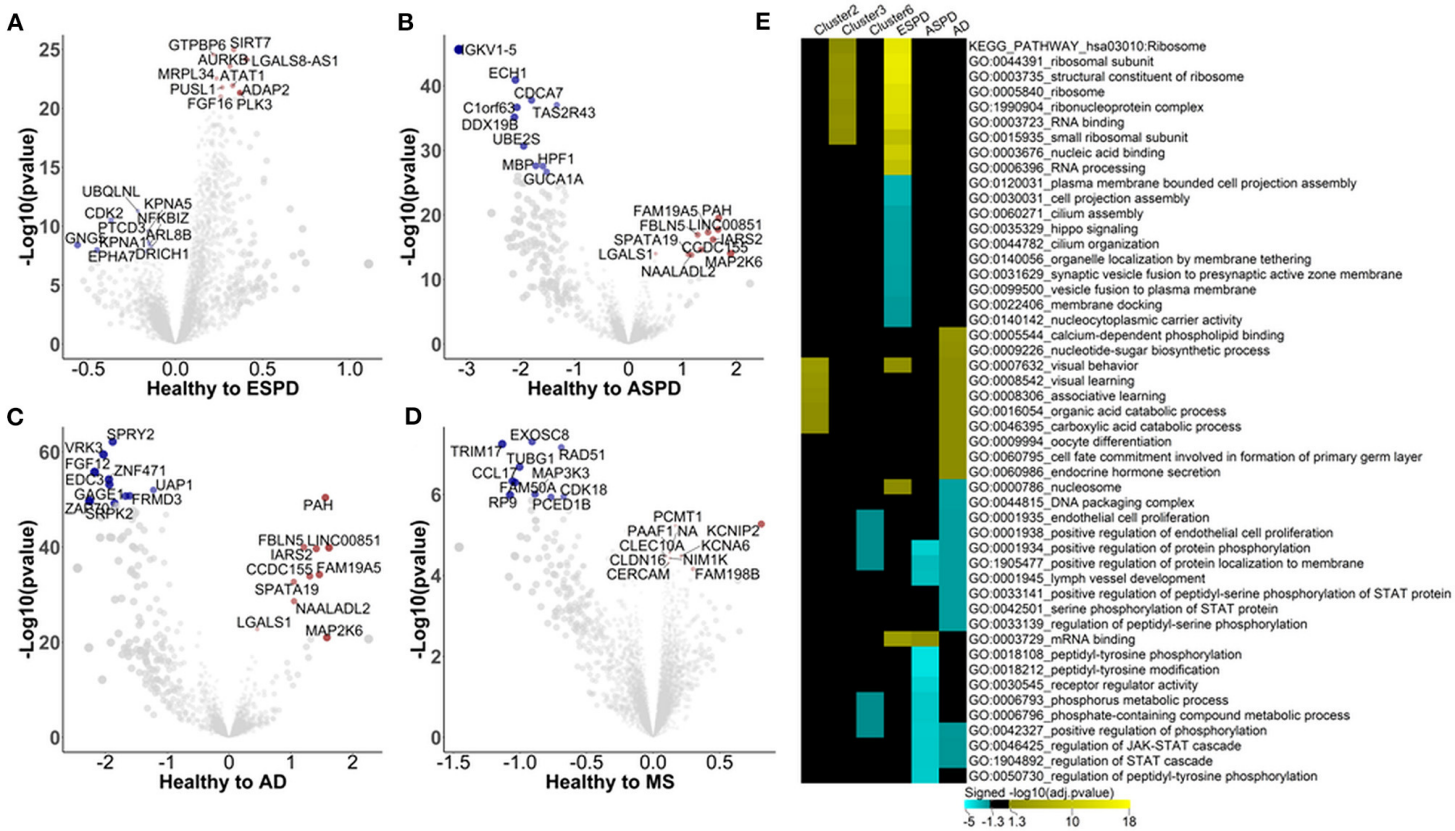
Autoimmunomic signatures of neurodegnerative diseases identified by linear modeling. In total, serum levels of IgG autoantibodies against 9,256 human antigens in 103 patients with ESPD, 24 patients with ASPD, 46 patients with AD, and 30 patients with MS as well as their corresponding age- and gender matched healthy controls were analyzed using linear modeling. Volcano plots representing changes within the autoimmunome in ESPD **(A)**, ASPD **(B)**, AD **(C)**, and MS **(D)**. Dot sizes are proportional to the product of –log10(*p*-value) and disease effect (beta of the linear model). **(E)** Visualization of pathways significantly enriched for aging- and disease-associated autoantibodies identified by linear modeling. Autoantibodies upregulated and downregulated were analyzed separately. Top 10 significantly enriched gene ontology terms per condition per disease are represented, and aging related clusters are used as reference.

The strong effect of ESPD on the autoimmunome encouraged us to further explore the autoimmunomic signatures of age-related neurodegenerative diseases including ESPD, ASPD, AD, and MS. We retrieved autoantibody profiling data from 103 patients with ESPD, 24 patients with ASPD, 46 patients with AD, and 30 patients with MS, and compared them with equal numbers of age- and sex-matched healthy individuals. As compared to their corresponding controls, ESPD, ASPD, AD, and MS are significantly (*P*_adjusted_ < 0.05) associated with 2,723, 1,154, 1,571, and 236 autoantibodies, respectively (Figures 3A–D, Supplementary Table 8).

**Figure 4:**
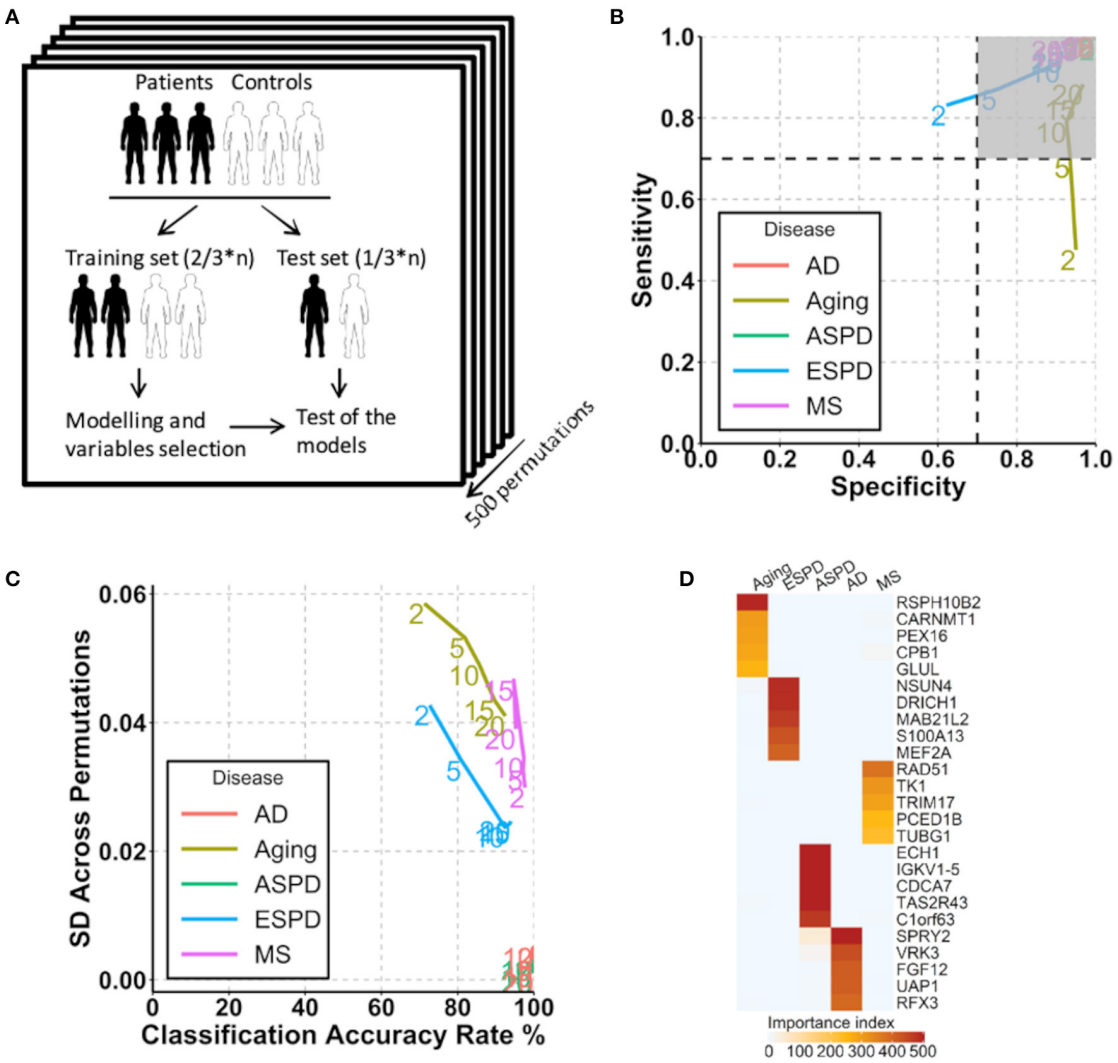
Prediction of aging and age-related diseases using autoantibodies. **(A)** Strategy for modeling and autoantibody selection for the prediction of aging and neurodegenerative diseases. The mean classifications accuracy rate, sensitivity, and specificity were calculated across the 500 resampled test data sets. **(B)** Path plot of sensitivity and specificity for models includes the 2 to 20 top selected autoantibodies for aging and neurodegenerative diseases. The numbers of autoantibodies are indicated by the numbers in the lines within the plot. Gray quadrant indicates the area of the figure in which sensitivity and specificity are higher than 0.7. **(C)** Path plot of the accuracy rate of classification and its SD across 500 permutations for models includes the top 2 to 20 selected autoantibodies for aging and neurodegenerative diseases. A small SD across permutations (close to 0) demonstrates a high stability of the model. **(D)** Heatmap of importance index of top 5 selected autoantibodies in aging and neurodegenerative diseases.

Enrichment analysis shows that autoantigens targeted by antibodies unregulated in ESPD were significantly enriched in GO terms of ribosome, RNA binding, translation, mitochondrial ribosome, and mitochondrial translation (Supplementary Table 9). Intriguingly, these GO terms were also associated with cluster 3 autoantibodies which increase consistently with age in healthy individuals (Figure 3E), suggesting a link between aging and ESPD. As compared to upregulated autoantibodies, enrichment for autoantigens targeted by antibodies downregulated in ESPD was much less prominent, with a significant enrichment e.g., in GO terms of cell projection assembly and hippo signaling. Unexpectedly, the autoimmunomic signature of ASPD differed considerably from that observed in ESPD. Autoantigens targeted by autoantibodies upregulated in ASPD were only significantly enriched in terms of mRNA binding (*P*_adjusted_ = 0.02), while those downregulated in ASPD were enriched in peptidyl-tyrosine phosphorylation, peptidyl-tyrosine modification, and receptor regulator activity which are also associated with aging (Figure 4D, Supplementary Table 9). AD, another age-related neurodegenerative disease, was also associated with autoantibodies related to aging. Many GO terms associated with autoantibodies increased during aging, including visual behavior, visual learning, associative learning, fatty acid oxidation and carboxylic acid. Furthermore, catabolic processes were also associated with upregulated autoantibodies in AD (Figure 3E, Supplementary Table 9). However, at least som GO terms associated with autoantibodies decreased with age in healthy subjects, such as endothelial cell proliferation and protein phosphorylation, found to be down-regulated in AD compared to healthy controls (Figure 3E, Supplementary Table 9). As an autoimmune-mediated neurodegenerative disease, MS was associated with autoantibodies which were not significantly enriched in any GO terms (Supplementary Table 9).

### Autoantibodies as Predictive Parameters for Aging and Age-Related Neurodegenerative Diseases

Given the strong association of the autoimmunome with aging and age-related diseases, we investigated whether autoantibodies could be suitable markers for the prediction of ESPD, ASPD, AD and MS. The term “prediction” is here in the context of probability theory and not in the sense of a medical prognosis. To this end, patients and their corresponding healthy controls were randomly divided into two subgroups, where two thirds of the subjects were used to build a predictive model that was validated on the remaining one third of individuals (Figure 4A). A corresponding analysis was performed to predict aging by categorizing healthy individuals into groups of members <65 years of age (status “young”) and ≥65 years of age (status “old”). The old-age threshold was defined as 65 years for two reasons. On the one hand, the term “elderly” has been conventionally defined as a chronological age of 65 years old or older (Sabharwal et al., 2015). On the other hand, it has been shown that healthy subjects over 65 years of age have higher number of detectable autoantibodies than those below 65 years (Nagele et al., 2013). Using the top 2 to 20 selected autoantibodies, we calculated sensitivity and specificity of each prediction model (Figure 4B).

Notably, autoantibodies were extremely accurate markers for the prediction of ASPD and AD, with sensitivity/specificity/accuracy of 100%/100%/100% in all predictions (Figure 4B). Furthermore, the SD across permutations indicated that prediction of ASPD and AD were stable (Figure 4C). Among 500 permutations, only 125 and 66 autoantibodies were used at least once for the prediction, suggesting a small proportion of the autoantibodies which differ considerably between the two diseases and their controls. The top five autoantibodies enabling a prediction of ASPD were enoyl-CoA hydratase 1(ECH1), immunoglobulin kappa variable 1-5 (IGKV1-5), cell division cycle associated 7 (CDCA7), taste 2 receptor member 43(TAS2R43) and DEAD-box helicase 19B (DDX19B) (Figure 4D, Supplementary Table 10). By contrast, prediction of AD was possible by the use of sprouty RTK signaling antagonist 2(SPRY2), vaccinia related kinase 3(VRK3), fibroblast growth factor 12 (FGF12), UDP-N-acetylglucosamine pyrophosphorylase 1(UAP1), and regulatory factor X3 (RFX3). Although less stable and accurate than ASPD and AD, MS and ESPD could also be predicted by autoantibodies with high and stable accuracies (Figures 4B,C, Supplementary Table 10). By contrast, the prediction of aging was largely dependent on the number of autoantibodies used, where the prediction with top 20 autoantibodies led to an acceptable accuracy (sensitivity/specificity/accuracy, 88%/97%/90%) but the prediction with top 2 autoantibodies led to a low accuracy (sensitivity/specificity/accuracy, 48%/95%/71%) (Figures 4B,C, Supplementary Table 10).

**Figure 5:**
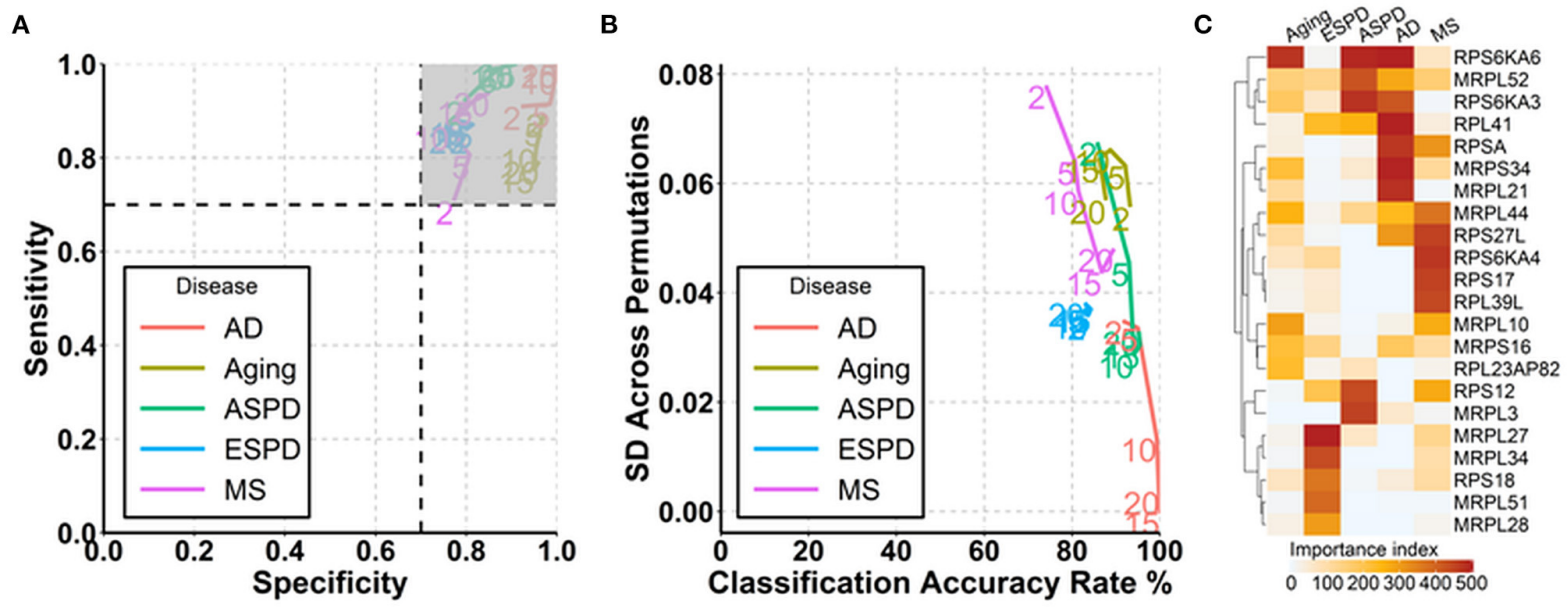
Prediction of aging and age-related diseases using autoantibodies against ribosomal proteins. **(A)** Path plot of sensitivity and specificity for models includes the 2 to 20 top selected autoantibodies against ribosomal proteins for aging and neurodegenerative diseases. **(B)** Path plot of the accuracy rate of classification and its SD across 500 permutations for models includes the top 2 to 20 selected autoantibodies against ribosomal proteins for aging and neurodegenerative diseases. **(C)** Heatmap of importance index of top 5 selected autoantibodies against ribosomal proteins in aging and neurodegenerative diseases.

Since autoantibodies that increase progressively and consistently with age are associated with ribosomes and ribosomal proteins are suggested to be associated with aging (Kaushik and Cuervo, 2015; Steffen and Dillin, 2016), we next determined whether autoantibodies against ribosomal proteins could be used for the prediction of aging and age-related diseases. Out of 146 autoantibodies against ribosomal proteins, 145, 144, 134, 102, and 83 were used at least once for the prediction of aging, ESPD, MS, ASPD, and AD, respectively, across 500 permutations (Supplementary Table 11). Noticeably, high and stable prediction accuracy was observed in AD (91–100%), followed by ASPD (85–95%), aging (86–93%), ESPD (82–84%), and MS (74–89%) (Figures 5A,B). Regarding the individual autoantibody level, IgG against ribosomal protein S6 kinase A6 (RPS6KA6) was the most frequently selected autoantibody for the prediction of AD, ASPD and aging (Figure 5C, Supplementary Table 11).

## Discussion

Biological aging is a progressive and inevitable process featured by declined functions of cells and tissues/organs. Previous studies have identified transcriptomic, metabonomic, methylatomic, and proteomic signatures for aging (Jylhava et al., 2017). In this study, we reanalysed the autoantibody profile data of healthy subjects and patients with ESPD, ASPD, AD, and MS from GEO database (DeMarshall et al., 2015). Different to DeMarshall's study in which the identification of diagnostic autoantibodies for disease was aimed by examining differentially expressed autoantibodies between patients and controls, we here intended to identify and characterize autoimmunomic signatures of aging and age-related diseases and to search for predictive autoantibodies. Linear model regression revealed that autoimmunomic signatures of aging and age-related diseases are featured by an increased expression of IgG autoantibodies associated with learning, behavior, ribosome and translation. Moreover, DE-SWAN showed that autoantibodies in healthy subjects change in an undulating pattern during aging. In addition, by using elastic net algorithm we identified a different set of autoantibodies as the previous study (DeMarshall et al., 2015) as parameters for the prediction of age-related neurodegenerative diseases. To our knowledge, this study for the first time revealed that there are distinct patterns and/or waves of autoantibodies with aging and age-related neurodegenerative diseases.

Similar to aging-associated expression of plasma proteins (Lehallier et al., 2019), most age-associated autoantibodies change in an undulating pattern. Moreover, quantification of the changes in the autoimmunome revealed two major waves in the fourth and seventh decade of life, which overlaps in their time windows with the waves of changes in the plasma proteome (Lehallier et al., 2019). However, comparison between the waves of autoimmunomic changes with those of plasma proteomic changes revealed that there is only little overlap between antigens targeted by the changed autoantibodies and changed plasma proteins, suggesting that autoimmunomic alternations are not a consequence of the abnormalities in plasma protein expression. This notion is further supported by the association of waves of autoimmunomic changes with tissues/organs, where wave in the fourth decade of life is associated with brain and its function and that in the seventh decades of life is associated with various tumors. Given that dysregulation of homeostasis of tissue could be a trigger of autoimmunity (Petersen et al., 2017), the autoimmunomic changes might reflect the abnormalities in peripheral tissues.

Interestingly, the two major aging-related waves of autoantibody changes identified in healthy subjects were not observed in patients with ESPD. There are probably three reasons for the lack of the aging-related waves in patients. First, the neurodegenerative disease shows a strong impact on the autoantibody profile, potentially masking the effect of aging on the changes of autoantibodies. Second, unlike healthy control group, the starting age of ESPD group is at 37 years, which makes it challenging to detected the wave in the fourth decade of life, Third, patients with ESPD might have different baselines and waves of autoantibody changes from the healthy subjects.

Maybe the most important feature of the autoimmunomic signatures of aging and age-related diseases is that they are associated with brain and its function. Autoantibodies which increase considerably at the third and fourth decades of life are significantly enriched in the biological processes of learning and behavior. In line with this finding, the wave of autoimmunomic changes at the fourth decade of life is associated with hippocampus, learning and behavior. Furthermore, learning and behavior are also associated with autoantibodies whose expression is increased in AD and ESPD. It is widely accepted that oxidative stress increases during aging and neuronal cells are highly sensitive to oxidative stress (Finkel and Holbrook, 2000), leading to the elevation of oxidized neuron proteins (Wang and Michaelis, 2010). Beside oxidized proteins, other proteins abnormalities such as misfolding and aggregation are also hallmarks in neuron cells during aging and neurodegeneration (Perluigi et al., 2014; Walther et al., 2015). Although the protein abnormalities are principally physiological features of aging, excessive levels of such abnormalities are associated with age-related neurodegenerative disorders, including AD and PD (Chen et al., 2012). Therefore, it is conceivable to speculate that the association of aging and age-related diseases with autoantibodies targeting neurological antigens might reflect proteins abnormalities in neuronal cells. Although it is not known whether these autoantibodies are of physiological or pathological relevance, they reflect the proteins changes in the neuron cells and thus could be potential markers for aging and age-related diseases.

Only a small portion of autoantibodies increase with age consistently in a linear manner. A common key feature of those autoantibodies is that their antigens are predominantly expressed in ribosomes. Although autoantibodies against ribosomal proteins have been observed patients with systemic lupus erythematosus for more than 30 years (Choi et al., 2020), the association of those autoantibodies with aging and age-related disease has not been reported. Given that ribosomes are the factories tasked with protein synthesis and thus regulate proteostasis, it is not surprising that ribosomes play a critical role in aging. Moreover, it has been demonstrated that deficiency of specific ribosomal proteins could significantly affect the life span in many experimental organisms (Hansen et al., 2007; Steffen et al., 2008; Zid et al., 2009; Houtkooper et al., 2013). In humans, a genetic polymorphism within the mitochondrial ribosomal protein L23 (MRPL23) gene has been shown to be associated with cognitive aging (Moulton, 2018). Biogenesis of ribosomes is a complex process which encompasses a complicated series of events including the synthesis and processing of ribosomal RNAs, assembly of ribosomal proteins, transport to the cytoplasm and association of ribosomal subunits (Turi et al., 2019). Our study shows that autoantibodies against ribosomal proteins consistently increase with age and are associated with age-related neurodegenerative diseases. There are two possible explanations for this phenomenon. At first, abnormalities in ribosome biogenesis during aging and development of age-related neurodegenerative diseases may lead to the generation of autoantibodies against ribosomal proteins. This notion is supported by findings that aging is associated with aberrant ribosome biogenesis, including accumulation of DNA damage (Flach et al., 2014) and progressive decrease in the expression of ribosomal proteins (Jung et al., 2015). Furthermore, deregulated ribosome biogenesis is observed in age-related neurogenerative diseases. For example, it has been reported that the early phase of AD is associated with ribosome dysfunction including a decreased rate and capacity for protein synthesis, decreased ribosomal RNA and tRNA levels, and an increased RNA oxidation (Ding et al., 2005). In addition, downregulation of ribosome biogenesis has also been observed in patients with PD (Rieker et al., 2011; Taymans et al., 2015). The second possibility is that the abnormalities occur first in the immune system with aging, which impacts the ribosome biogenesis thereby generating autoantibodies against ribosomal proteins. Moreover, this study demonstrates that autoantibodies can be used as prediction markers for aging and age-related diseases. Even if the analysis is limited to autoantibodies against ribosomal proteins only, it still allows a high and stable accuracy in the prediction of AD and PD. Taken together, autoantibodies against ribosomal proteins are associated with and can be used as prediction markers for aging and age-related diseases, which in line with the fact that ribosome is essentially involved in the physiological and pathological changes during aging.

It is necessary to mention that for aging and each disease, only one cohort was available to explore the autoimmunomic signatures, and a validation of our findings in an independent dataset was not possible. Moreover, numbers of healthy individuals and patients are relatively small, which limits the statistical power and might lead to type II error. Particularly, this may critically impact the predictive analyses where the cohorts are divided into two subsets for training and test, respectively. Therefore, further investigations with high power and longitudinal components are required to further substantiate the findings of this study.

In conclusion, this study for the first time uncovers autoimmunomic signatures for aging and age-related neurodegenerative diseases. Identification of autoantibodies that associated with aging or age-related diseases will likely improve the prediction of aging and the diagnosis of the diseases. Moreover, further examining the function of those autoantibodies might open new therapeutic strategies for treating age-related neurodegenerative conditions.

## Data Availability Statement

Publicly available datasets were analyzed in this study. This data can be found here: https://www.ncbi.nlm.nih.gov/geo/query/acc.cgi?acc=GSE62283.

## Author Contributions

XY conceived the study. JY retrieved and analyzed data. All authors wrote the manuscript.

## Conflict of Interest

The authors declare that the research was conducted in the absence of any commercial or financial relationships that could be construed as a potential conflict of interest.
