# Supplementary material for: Autoimmunomic Signatures of Aging and Age-Related Neurodegenerative Diseases Are Associated With Brain Function and Ribosomal Proteins
Source: Front Aging Neurosci. 2021 May 28;13:679688. doi: 10.3389/fnagi.2021.679688 (PMC8192960; doi:10.3389/fnagi.2021.679688)
Supplement: Supplementary file 2 [file Data_Sheet_1.PDF]

Suppelmentary information

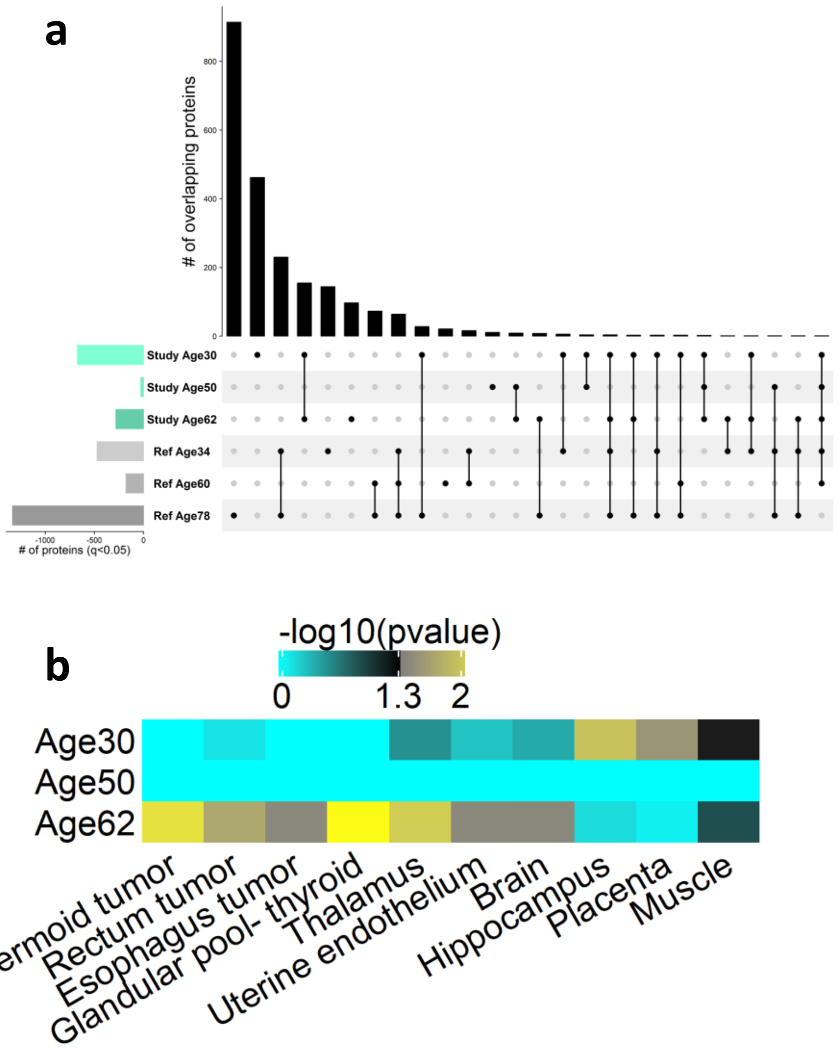

Supplementary figure 1. Comparison between waves within the autoimmunome and the plasma proteome of aging. Autoantibodies significantly changed in the three waves across the lifespan (age30, 50 and 62) were identified in this study by using DE-SWAN, while plasma proteins changes in three waves (age 34, 60 and 78) were identified in a previous study (Lehallier et al., 2019). **a**, Intersections between the autoimmunomic and plasma proteomic waves visualized by Upset plot. **b**, Visualization of UP-TISSUE significantly enriched for aging associated autoantibodies identified by DE-SWAN at age 30, 50 and 62.

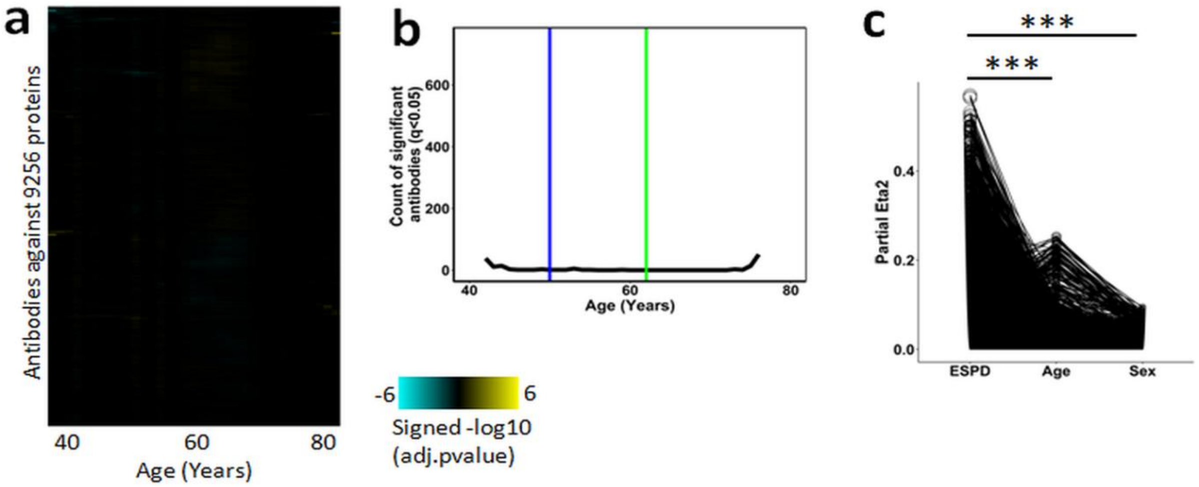

Supplementary figure 2. Waves of autoimmunomic changes with age in patients with ESPD. **a**, Changes in autoantibody expression during aging in the early stage of Parkinson's disease (ESPD) characterized by DE-SWAN. **b**, Number of autoantibodies differentially expressed ($P_{adjusted}$ <0.05) during aging in ESPD patients identified by DE-SWAN. Blue and green lines indicate peak of waves of aging-ralated autoantibodies at age of 50 and 62 , respectively, which were identified at healthy controls (Figure 2b). **c**, Relative percentage of variance explained by ESPD, age and sex, where Partial Eta2 is calculated for ESPD, age and sex. Values for each autoantibodies are connected by edges.
